# Self-Sensing Carbon Nanotube Composites Exposed to Glass Transition Temperature

**DOI:** 10.3390/ma13020259

**Published:** 2020-01-07

**Authors:** Sung-Hwan Jang, Long-Yuan Li

**Affiliations:** 1 Civil and Environmental Engineering, School of Engineering, Hanyang University ERICA, Ansan, Gyeonggi-do 15588, Korea; 2 Civil and Coastal Engineering, School of Engineering, University of Plymouth, Plymouth, Devon PL7 8AA, UK; long-yuan.li@plymouth.ac.uk

**Keywords:** carbon nanotubes, epoxy, electrical-mechanical behavior, self-sensing, glass transition temperature

## Abstract

This paper reported the effect of high temperature on the electro-mechanical behavior of carbon nanotube (CNT) reinforced epoxy composites. CNT/epoxy composites were fabricated by dispersing CNTs in the epoxy matrix using a solution casting method. Electrical conductivity measurements obtained for the CNT/epoxy composites indicated a steadily increasing directly proportional relationship with CNT concentration with a percolation threshold at 0.25 wt %, reaching a maximum of up to 0.01 S/m at 2.00 wt % CNTs. The electro-mechanical behavior of CNT/epoxy composites were investigated at a room temperature under the static and cyclic compressive loadings, resulting that the change in resistance of CNT/epoxy composites was reduced as increasing CNT concentration with good repeatability. This is due to well-networked CNTs conducting pathways created within the solid epoxy matrix observed by scanning electron microscopy. Temperature significantly affects the electro-mechanical behavior of CNT/epoxy composites. In particular, the electro-mechanical behavior of CNT/epoxy composites below the glass transition temperature showed the similar trend with those at room temperature, whereas the electro-mechanical behavior of CNT/epoxy composites above the glass transition temperature showed an opposite change in resistance with poor repeatability due to unstable CNT network in epoxy matrix.

## 1. Introduction

In recent years, carbon nanotubes (CNTs) have attracted considerable interest for many industrial applications [1,2,3]. CNTs possess excellent mechanical, electrical, electronic, optical, chemical and thermal properties, which, when combine with their very high aspect ratio and large surface area, have made them an excellent candidate for smart composite materials [4,5,6,7]. In this context, CNT reinforced composite materials have been investigated for smart composite applications such as for gas detection [8], structural integrity self–sensing [9] and actuators [10]. Unlike conventional materials, CNT reinforced composites have many advantages such as being relatively light in weight, having good corrosion resistance and waterproofing, and with the potential for self-sensing applications. For example, Ku-Herrera et al. [11] studied the strain sensitivity of nanocomposites containing a small amount of CNTs. They found that the mechanical properties of the nanocomposite were improved during compression testing reaching a maximum value, in terms of gauge factor, at 0.3 wt % of CNT content. Ayatollahi et al. [12] investigated the effect of CNT concentration on the mechanical and electrical properties of CNTs/epoxy nanocomposite. They found that both of the mechanical and electrical properties were improved especially for a 1.0 wt % CNTs. 

The strain sensing capability of CNT nanocomposites is considered to be a key requirement for future structural health monitoring techniques and applications. To overcome these problems and requirements, some studies have tried to develop novel self-sensing nanocomposites for these types of applications [13,14,15,16,17]. Bouhamed et al. [6] fabricated a CNT/epoxy nanocomposite and focused on the behavior of the nanocomposite under strain applications. The results showed a higher changing in resistance happen at low CNTs concentration and the strain sensitivity at this content was 14.19. In addition, Dinh et al. [18] investigated the behavior of adding CNTs to epoxy when fabricating a self-sensing nanocomposite structure. They found that for an applied and stepped changed cyclic pressure, the electrical resistance of the structure increased with increasing pressure and decreased with decreasing pressure. During room temperature, testing the nanocomposite structure remained stable and showed excellent reproducible results. Shen et al. [7] studied changes in electrical resistance of self-sensing CNTs in epoxy nanocomposite during compression testing. They observed that when the compression load increased the electrical resistance of the nanocomposite decreased, thus, indicating that it exhibited a measurable piezoresistive effect.

The effect of temperature on CNT reinforced composites has been studied for different filler-resin composites. Gojny and Schulte [19] investigated the effect of multi-walled CNTs on the thermo-mechanical properties of MWCNT/epoxy composites and found that increasing concentrations of MWCNTs as well as functionalizing MWNCTs leads to an increase of the glass transition temperature with higher interfacial interaction between the CNT and the polymer matrix. Godara et al. [20] reported that CNT reinforced epoxy/carbon fiber composites showed significant decrease in thermal expansion and increase in fracture toughness Mode-1. However, although previous researchers have investigated material properties of CNT reinforced composites, they did not consider the effect of glass transition temperature on the electro-mechanical behavior of CNT reinforced composite materials, which is critical parameter for real structural health monitoring application, and is described here for the first time. This omission leaves a gap in our understanding of the phenomena, since we know that when the temperature increases, the stiffness of the combined matrix and CNTs is likely to decrease due to its nature of polymer. Under these conditions, the electrical resistance of the CNTs themselves may also change due to unstable CNT network. The extent of these changes remains largely unknown. Therefore, in this study, we investigated the temperature dependence of the electro-mechanical behavior of CNT/epoxy composites.

## 2. Materials and Methods 

A filler material used for the CNT/epoxy composite was high purity (>95.0 wt %) and chemical vapor deposition grown multi-walled carbon nanotubes from US Research Nanomaterials Inc. (Houston, TX, USA). The average diameter and length of CNTs were 4–10 nm and 50 µm, respectively. A low viscosity epoxy resin type (IN2 Epoxy Infusion Resin) combined with a hardener (AT30 slow) were obtained from Easy Composite (Stoke-on-Trent, UK). The viscosity of the matrix resin was 200–450 mPas and a pot life was 80–100 min at a room temperature. The solvent used for dispersion of CNTs was high purity of acetone (> 95%) supplied by Acros Organics Ltd (Loughborough, UK). 

The CNT/epoxy composites were fabricated by uniformly dispersing the CNTs in the epoxy matrix as described in authors’ previous studies [21,22,23]. Firstly, various concentrations of the CNTs (0–2.5 wt %) were weighed out and mixed in 60 mL of acetone in a beaker. A high intensity and high frequency horn-type ultrasonicator (BR-20MT-10L, 1000 W) was used to ensure full and uniform dispersion of the CNTs in the epoxy resin. This approach was used to negate the van der Waals forces that exist between the CNTs and which tend to make them clump together. In this study, the ultrasonicator operated in an ice bath in the pulsed mode (45 s on and 15 s off) for 30 min to minimize any overheating effect. Following sonication of the CNTs, a weighed amount of epoxy resin was added to the mixture and dispersed again using the ultrasonicator for another 5 minutes. The fully dispersed mixture was then placed in an oven at 70 °C for 24 h to evaporate the acetone. Once evaporation was completed, the hardener (AT30) was added, with a mix ratio of 100:30, and thoroughly mixed for a further 5 min. The mixture was then degassed for 20 min in a vacuum chamber to remove any damaging air bubbles from the mixture. The mixture produced in this way was then cast in a mold to produce the desired specimen configuration needed for testing. Finally, the mixture was cured 24 h at room temperature and then for a further 24 h post cure at 60 °C to ensure the full completion of the cure process. All samples were cut into 25 mm diameter cylinder with 20 mm in height for the electro-mechanical response of CNT/epoxy composites.

**Figure 1:**
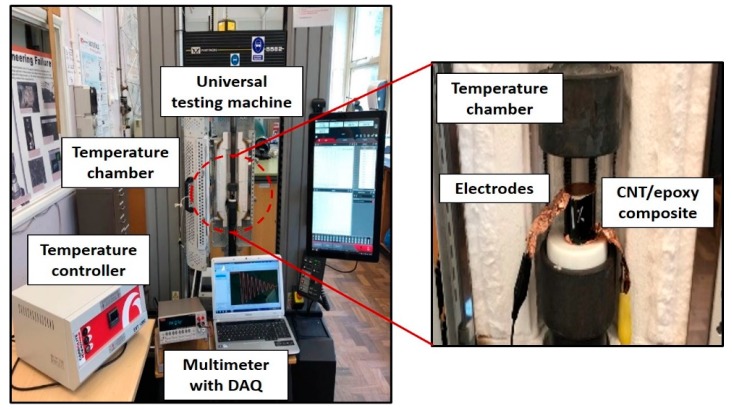
Electro-thermo-mechanical test set-up.

A fracture surface of the specimen was observed using a scanning electron microscope (JEOL JSM-7001F, Tokyo, Japan) at 30 kV to assess the dispersion of CNTs in the matrix. The samples were coated with a thin gold film using sputter coating (QUORUM-Q150TES, Laughton, UK) for 10 min. For the electrical conductivity, two different multimeters (Keithley 6517B for high resistance and Keithley 2700 for nominal resistance) was used to record resistance. To minimize measurement errors due to the contact resistance between the tip of test probe, both electrodes of the specimen was painted with high purity silver, eliminating an issue for the contact resistance to use two-probe method. Two-probe method was applied for the measurement of the electrical resistance for our specimens because of simple experiment. The electrical conductivity (*σ*) of samples was calculated using Equation (1).

(1) $$\sigma =\frac{L}{AR}$$

where *R* is the electrical resistance of the sample, *A* and *L* are a cross-sectional area and the length of the samples, respectively. The compression testing of the specimens was carried out using a universal testing machine (Instron 5582, Norwood, MA, USA). In order to investigate the effect of temperature on the electromechanical behavior of the specimens, the static and cyclic compressive loading was applied to the specimens at a displacement control of 0.5 mm/min. Temperature was controlled by placing the specimens in a heater box, as shown in Figure 1. Prior to testing, the specimens were hold for 1 h for uniform temperature distribution. During the static and cyclic loading, the change in resistance of the specimens was recorded using Keithley 2700 with 7700 data acquisition (DAQ) system.

## 3. Results

**Figure 2:**
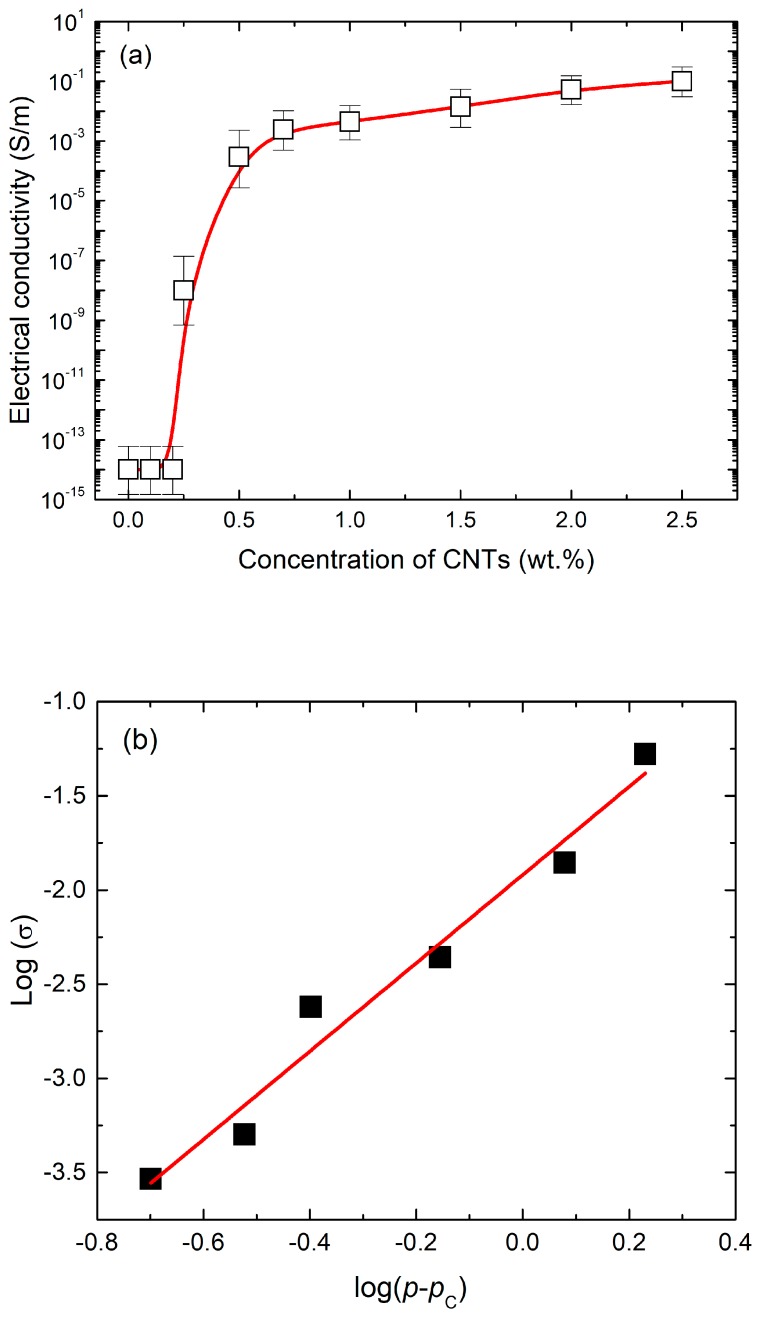
(**a**) Electrical conductivity and (**b**) curve fitting of CNT/epoxy composites.

**Figure 3:**
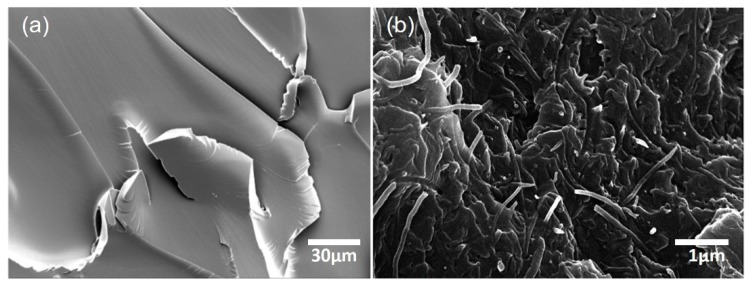
Scanning electron microscope (SEM) images of (**a**) pristine epoxy and (**b**) CNT/epoxy composite (2.0 wt %).

Figure 2a showed the electrical conductivity for the CNT/epoxy nanocomposites as a function of CNT concentrations. Pure epoxy is non-conductive materials with 1 × 10^−14^ S/m. A significant increase in the electrical conductivity was observed when the concentration of CNTs increased from 0.2 wt % to 0.5 wt %, indicating a percolation threshold. Thereafter, the electrical conductivity gradually increased up to 2.5 wt % CNTs. The percolation threshold, i.e., the minimum CNT content in the matrix after which no significant change in the electrical conductivity is observed, occurred at around 0.25 wt % CNTs. The observed increased in electrical conductivity of the CNT/epoxy composite is due to a well-developed CNT network structure (conducting pathways) created within the matrix material as shown in Figure 3. The effectiveness of electron transfer between the CNTs is very highly dependent on this CNTs spacing distance. In this study, the maximum electrical conductivity of CNT/epoxy composite reached was 5.25 × 10^−2^ S/m at 2.5 wt % CNTs. The experimental data were also analyzed using a percolation theory, $\sigma =\sigma_{0}{\left(p-p_{c}\right)}^{t}$, $\sigma_{0}$ is a parameter depending on the electrical conductivity of CNT, p is the volume fraction of CNTs, $p_{c}$ is the volume fraction corresponding to the percolation threshold, t is the critical exponent, as shown in Figure 2b, where $p_{c}$ and t are 2.34 and 2.0, respectively. Note that the value of t obtained proves that the CNT networks are deformed as a three-dimensional aspect inside the nanocomposite matrix.

**Figure 4:**
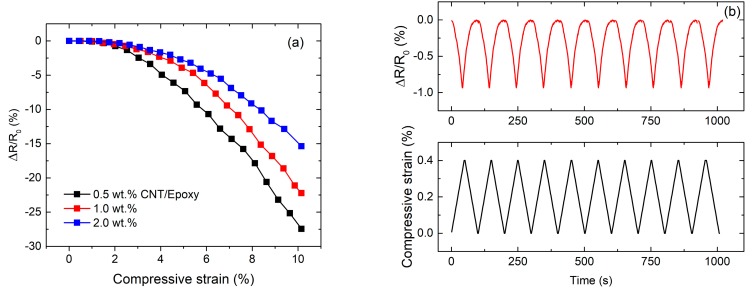

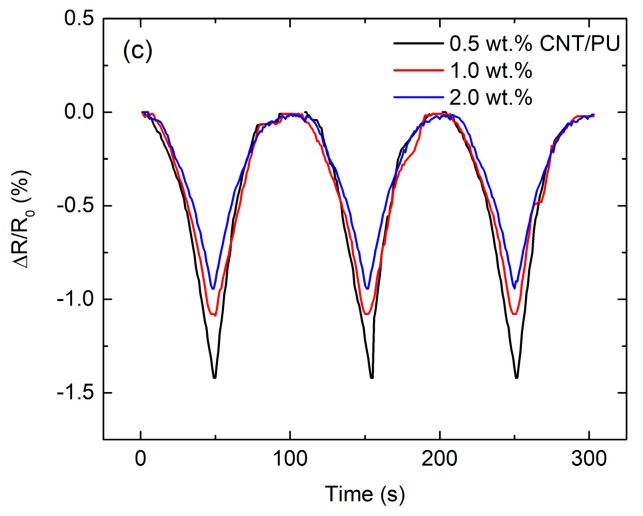
(**a**) Change in resistance of CNT/epoxy composites under static compressive loading; (**b**) electro-mechanical response of CNT/epoxy composites (2.0 wt %) under cyclic compressive loadings, (**c**) maximum peak at three cycles for all CNT/epoxy composites.

The electro-mechanical response of CNT/epoxy composites was investigated under both static and cyclic compressive loadings. Figure 4a showed the change in resistance of CNT/epoxy composites under the static compressive loadings at room temperature. It can be clearly seen that the resistance of all specimens decreased with increasing compressive loading with nonlinear behavior. Additionally, the piezo-resistive behavior of CNT/epoxy composites is similar to that observed by Yin et al. [24], which significantly depends on CNT concentration. A higher CNT content lead to a smaller resistance change during the compressive loadings. This could be due to the fact that increasing CNTs concentration reduces the inter-particle distance between adjacent CNTs, thus allowing the potential for greater possible contact between them under that compressive loading. Figure 4b,c showed the change in resistance of the CNT/epoxy composites under cyclic compressive loadings within elastic region at the room temperature. The resistance changes for the specimen showed excellent correspondence with the changes in the applied compressive strain. It was also observed that CNT/epoxy composite with higher CNT concentration provides lower strain sensitivity under the cyclic compressive loadings.

**Figure 5:**
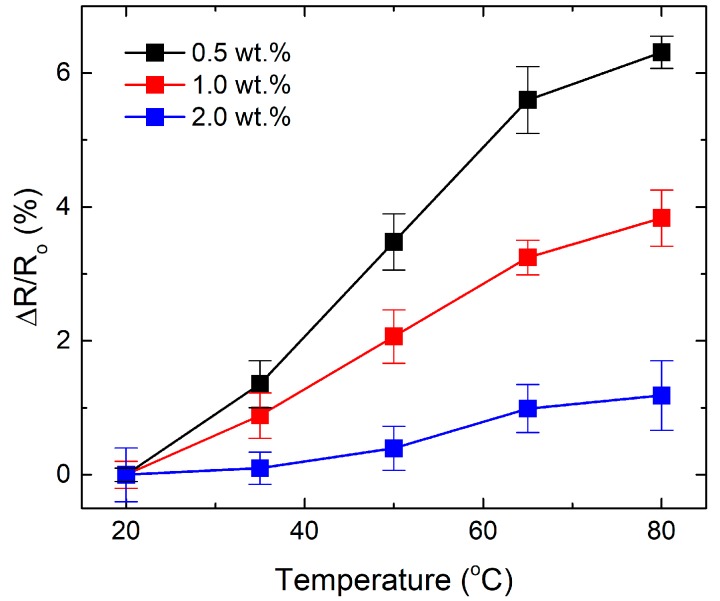
The effect of temperature on the change in resistance of CNT/epoxy composites at rest.

Temperature generally affects the materials properties of CNT reinforced composite [25,26,27]. Figure 5 showed the influence of temperature on the change in resistance of CNT/epoxy composites at rest. All CNT/epoxy composites presented a positive temperature coefficient, where the resistance of all samples increased at elevated temperature. The positive temperature coefficient for CNT/epoxy composites can be explained by the rearrangement of CNT network brought about by the volumetric expansion of epoxy matrix as the temperature rises. It can be noted that the change, with temperature, in the normalized resistance for the sample with the highest CNT concentration is smaller than for the low CNT. This behavior can be associated with the better stability of CNT network structure, associated with the higher CNTs concentrations, facilitating better electron transfer through the inter-particle channels [28].

**Figure 6:**
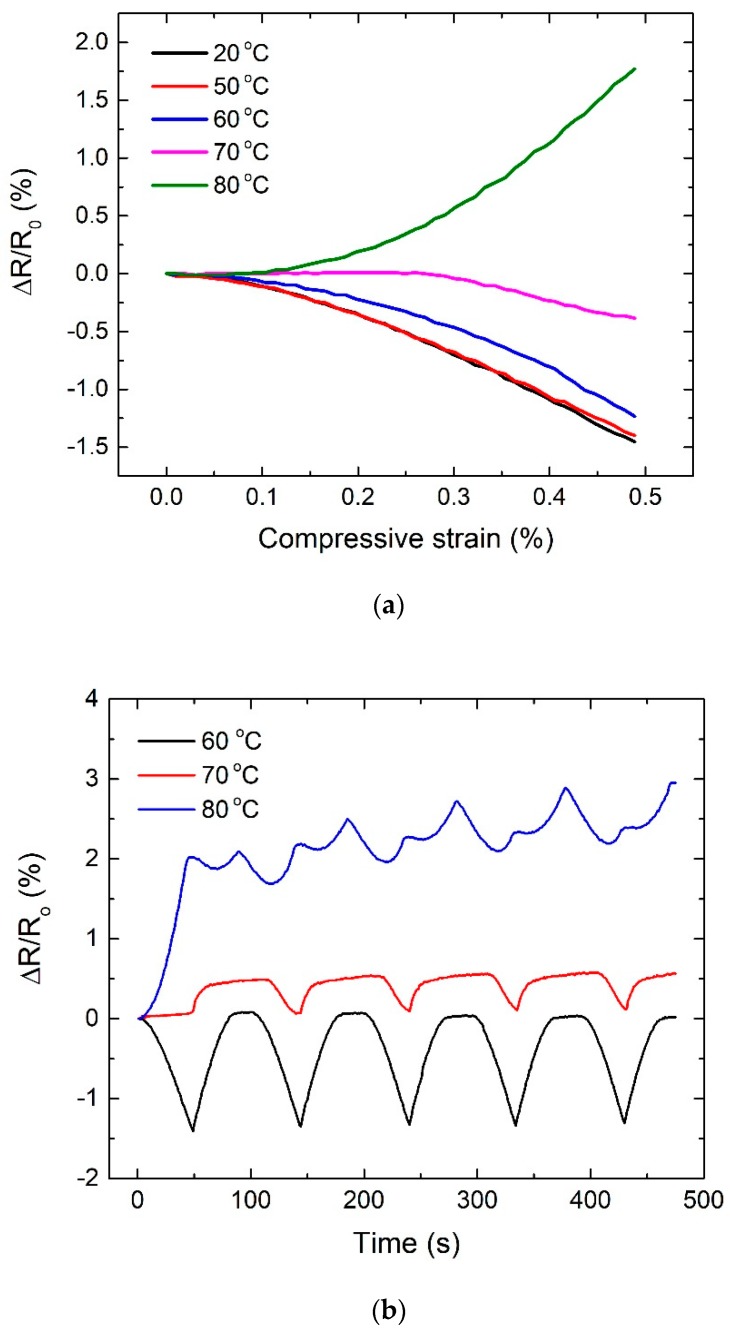
Electro-mechanical behavior of CNT/epoxy composites (1.0 wt %) under (**a**) static compressive loading and (**b**) cyclic compressive loading.

**Figure 7:**
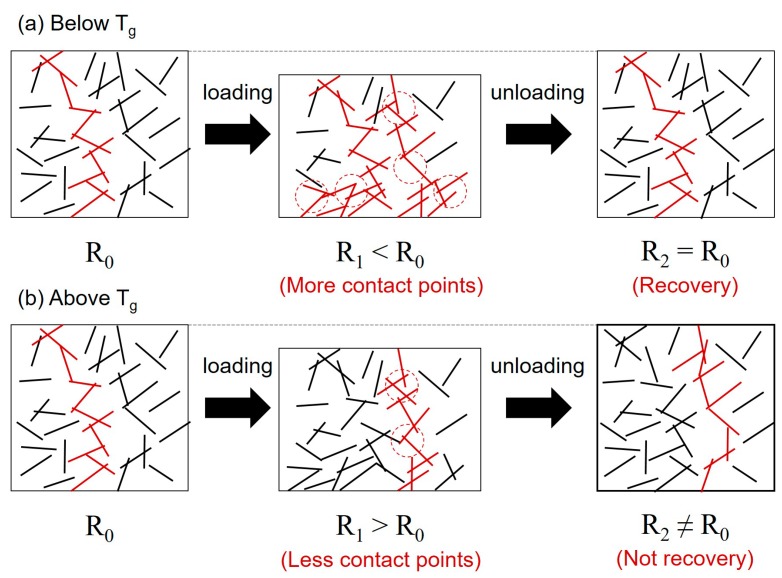
Schematic illustration of microstructure of CNT/epoxy composite (**a**) below the glass transition temperature and (**b**) above the glass transition temperature.

Although there has been much research on the effect of temperature on electrical properties of carbon nanotube reinforced composites at rest, there has been no research on the electro-mechanical behavior of those materials under the different temperatures. Therefore, we studied the electro-mechanical behavior of CNT/epoxy composite with 65 °C of the glass transition temperature [29] under static as well as cyclic compressive loadings. Figure 6a showed the change in resistance of 1.0 wt % CNT/epoxy composites under the static compressive loading at various temperatures. It was observed that the resistance behavior of samples varies with applied temperature. The specimens exposed to 20–60 °C showed the similar electro-mechanical behavior during the static compressive loading, whereas the specimen with 70 °C showed significant reduction in resistance compared to those with 20–60 °C and the specimen with 80 °C showed the increase in resistance which is an opposite trend with the samples with 20–60 °C. Figure 6b showed the electro-mechanical behavior of CNT/epoxy composites under the cyclic compressive loadings. For specimens tested between 20–60 °C, the resistance change observed remained remarkably consistent in nature during the cyclic compressive loadings. However, above this temperature range, those tested in 70 and 80 °C exhibited a different resistance response with poor repeatability, which is not applicable for repeatable sensing application. Considering their glass transition temperature (T_g_) of 65 °C, T_g_ significantly affects the electro-mechanical response of CNT/epoxy composites. It can be speculated, shown in the Figure 7, that the irreversible volumetric change of CNT/epoxy composite due to T_g_ would increase in the inter-distance between CNTs and thus regenerate CNT network, destroying conductive pathways and thus increasing resistance under the compressive loadings.

## 4. Conclusions

The present study investigated the electro-mechanical behavior of carbon nanotube reinforced epoxy composites at various temperature. It was found that temperature significantly affects the electro-mechanical behavior of CNT/epoxy composites. In particular, the CNT/epoxy composites exposed to above glass transition temperature showed an opposite and unrepeatable electro-mechanical behavior under the compressive loadings compared to those exposed to below glass transition temperature. CNT/epoxy composites may enable potential applications to sensors and sensor-integrated materials. However, the influence of temperature on the electro-mechanical behavior for CNT/epoxy composite must be considered.
